# A rare case report: fulminant myocarditis in neonatal lupus erythematosus

**DOI:** 10.3389/fcvm.2025.1538903

**Published:** 2025-07-01

**Authors:** Shujun Tan, Long Li, Hui Zhang, Adilai Dawuti, Nuerya Rejiafu

**Affiliations:** Neonatal Center, Xinjiang Hospital of Beijing Children's Hospital, Children's Hospital of Xinjiang Uygur Autonomous Region, Urumqi, China

**Keywords:** neonatal lupus erythematosus, fulminant myocarditis, case report, cardiac involvement, literature review

## Abstract

**Background:**

Neonatal lupus erythematosus (NLE) is a rare autoimmune disorder caused by the transplacental passage of maternal autoantibodies, such as anti-Ro/SS-A and anti-La/SS-B. It can affect multiple organ systems, resulting in various symptoms. Fulminant myocarditis, a severe inflammatory heart condition and uncommon complication of NLE, may have a favorable prognosis with timely diagnosis and proper treatment.

**Case presentation:**

We present a case of a one-month-old female infant with seizures, hypocalcemia, and a maternal history of Sjögren's syndrome, which are compatible with NLE. Further diagnostic assessments detected anti-nuclear antibodies, anti-SSA, and anti-RO52 antibodies. The infant also exhibited elevated cardiac biomarkers and a reduced left ventricular ejection fraction, pointing to fulminant myocarditis. Treatment with intravenous immunoglobulin and methylprednisolone led to remarkable clinical improvement and normalization of laboratory parameters within one year.

**Conclusions:**

This case highlights the rarity of fulminant myocarditis in neonatal NLE and emphasizes the critical role of early diagnosis and timely intervention. It also indicates the potential prognostic value of cardiac biomarkers in such cases.

## Background

NLE is an autoimmune disorder that arises from the transfer of damaging autoantibodies from the mother to her fetus through the placenta (1). The key antibodies implicated in this condition are anti-Ro/SS-A and anti-La/SS-B, whereas anti-U1 ribonucleoprotein (U1RNP) is identified with less consistency (2). NLE can influence various systems, manifesting symptoms across areas including the skin, heart, blood, nervous system, and liver (3). Typical presentations consist of skin rashes, cytopenia, increased aminotransferase levels, and, on occasion, heart block (3). Cardiovascular issues associated with neonatal lupus may involve transient arrhythmias, different severities of atrioventricular block (first, second, or third degree), dilated cardiomyopathy, and endocardial fibroelastosis (4). Of these, atrioventricular block is the most common cardiac complication related to neonatal lupus, whereas heart fibroelastosis occurs far less frequently. Fulminant myocarditis is an uncommon yet serious inflammatory heart condition that can pose serious risks to life (5, 6). This condition usually progresses rapidly, exhibiting sudden onset and significant decline within a period as short as two weeks or even just a few days (7). Less commonly, fulminant myocarditis may also be triggered by other infectious agents and various autoimmune disorders. Despite the exceptionally high early mortality rate associated with fulminant myocarditis, the outlook for the long term tends to be favorable once the initial at-risk phase has been surpassed (8).

## Case presentation

In this case report, we present a patient diagnosed with NLE and fulminant myocarditis. A one-month-old baby girl was admitted to the hospital due to persistent limb shaking for five days. She was delivered via cesarean section at 38 weeks and 5 days of gestation, with a birth weight of 2,900 g. The infant was fed a combination of breast milk and formula, and there was no reported history of oxygen deprivation or asphyxia during pregnancy or delivery. Prior to admission, the patient exhibited tonic-clonic seizures, characterized by upward deviation of the eyes, myoclonus of the upper limbs, and extension of the lower limbs, which resolved spontaneously despite multiple recurrences. The electrolyte test revealed a calcium level of 1.1 mmol/L (normal range: 2.25–2.75 mmol/L).

With calcium supplementation and sedative treatment using phenobarbital, the patient's condition improved. On the fourth day of hospitalization, from the first seizure (5 days prior to admission) to the onset of cardiac symptoms (on the fourth day of admission), her heart rate increased to 220 beats per minute. The electrocardiogram revealed arrhythmias, including both ventricular and supraventricular tachycardia, as well as ST-T changes. Consequently, she was transferred to the NICU, where the administration of lidocaine normalized her heart rate. Further investigation disclosed that the mother had Sjögren's syndrome. A comprehensive ANA spectrum test was conducted on the child on the fifth day after admission, which included indirect immunofluorescence (IIF) using HEp-2 cell substrate for ANA and enzyme-linked immunosorbent assay (ELISA) for quantitative detection of anti-SSA and anti-RO52 antibodies. The results were positive for anti-nuclear antibody (++), anti-SSA antibody (+++), and anti-RO52 antibody (+++). Additionally, the B-type brain natriuretic peptide concentration was greater than 5,000 pg/ml (normal range: 20–120 pg/ml), the troponin-I concentration was 16.56 ng/ml (normal range: 0.1–0.2 ng/ml), and the high-sensitivity troponin-T concentration was 3,546 pg/ml (normal range: 0.02–0.13 pg/ml). Cardiac ultrasound indicated a left ventricular ejection fraction of 31% (normal range: >55%). Given the critical condition of the child, a multidisciplinary case discussion led to the diagnosis of NLE combined with fulminant myocarditis. The patient was treated with intravenous immunoglobulin and methylprednisolone, resulting in a significant therapeutic effect. A follow-up conducted five months post-treatment indicated that the test results had gradually returned to normal. The high-sensitivity cardiac troponin T (hs.cTnT) measured at 15.75 pg/ml, troponin-I at 0.0112 ng/ml, ANA antibody was negative (−), and the antibody titer (IIF) was less than 1:100. The anti-SSA antibody was positive (+), and the anti-RO52 antibody was significantly elevated (++). We continued to monitor the child's progress until she reached one year of age, at which point all examination and laboratory results remained within the normal range.

## Discussion and conclusions

Not all children with NLE will develop the common clinical manifestations of connective tissue disease. The child in this case was found to be positive for autoantibodies after an immune test was performed following inquiry about the mother's pregnancy history of Sjögren's syndrome. This situation may cause children to miss effective preventive measures for NLE. Therefore, when a newborn has unexplained cardiovascular problems, possible immune diseases should be considered, the mother's medical history should be asked, and relevant auxiliary examinations should be performed in a timely manner.

In this research, we conducted a comprehensive review of existing literature concerning cardiac involvement in patients with NLE. This review drew upon case reports available in the Web of Science core database, spanning the period from 2010 to 2024, which are detailed in Table 1. Congenital heart block (CHB), which may encompass first-, second-, or third-degree atrioventricular block, is the most common cardiac manifestation in NLE (3). However, fulminant myocarditis is a relatively rare cardiac manifestation and only accounts for a small proportion of cases. This review focuses on case reports to highlight the rare cardiac manifestation of fulminant myocarditis. However, it has limitations. Fulminant myocarditis is a relatively rare cardiac manifestation, accounting for only a small proportion of cases. This review focuses on case reports to underscore the infrequent occurrence of fulminant myocarditis. However, it is important to acknowledge its limitations. Future studies should consider incorporating diverse research methodologies to attain a more comprehensive understanding, especially in larger patient cohorts. Additionally, other rare cardiac manifestations may include myocarditis, cardiomyopathy—often linked with endocardial fibroelastosis—and valvular irregularities that can lead to stenosis, regurgitation, and dysplasia resulting from inflammation and fibrosis (27).

**Table 1:** Literature review of cardiac involvement in NLE patients.

Year (reference)	Case	Cardiac manifestation
2011, Guettrot-Imbert et al. (9)	5	Isolated Mild Endocardial Fibroelastosis
2012, Leonibus et al. (10)	1	Isolated atrioventricular block
2012, Killen et al. (11)	1	Second-degree heart block
2013, Tetsuya et al. (12)	1	Three-degree heart block
2015, Tanriverdi et al. (13)	1	Third-degree heart block
2017, Maria-Teresa et al. (14)	1	Myocarditis, Flail tricuspid valve
2017, Kadivar et al. (15)	1	Moderate mitral regurgitation
2018, Li et al. (16)	2	Heart block
2019, Elnady et al. (17)	1	Right ventricular thrombus
2020, Habib et al. (18)	1	First-degree heart block
2020, Rumancik et al. (19)	1	Left bundle branch block and Cardiomyopathy
2021, A Pramono et al. (20)	1	Third-degree heart block
2021, Miselli et al. (21)	1	Coronary Involvement
2022, Jeffrey et al. (22)	1	Third-degree heart block and Flail tricuspid valve
2023, Liu et al. (23)	1	Third-degree heart block
2023, Maryam et al. (24)	1	Incomplete heart block
2023, Naela et al. (25)	1	Third-degree heart block
2023, Jain et al. (26)	1	Myocardial dysfunction

Fulminant myocarditis, while less common, is generally more severe than congenital atrioventricular block. Its primary trigger is viral infections (28); however, it can also be infrequently initiated by connective tissue disorders, such as NLE. The capability of high-sensitivity cardiac troponin T (hs-cTnT) to detect subclinical myocardial inflammation in infants who have been exposed to anti-Ro antibodies during gestation is still uncertain (29). In the case of our patient, a positive test result confirmed the presence of anti-Ro antibodies. Additionally, in relation to myocarditis, numerous biomarkers indicating myocardial damage and strain on the heart walls—such as cardiac troponin (cTn), B-type natriuretic peptide (BNP), and N-terminal prohormone of brain natriuretic peptide (NT-proBNP)—are often found at elevated levels (30, 31). In this specific case, we observed increased levels of BNP, high-sensitivity troponin, and troponin I. These heightened concentrations of biomarkers offer a thorough evaluation of the patient's myocardial state, signifying an active inflammatory response alongside myocardial injury. The simultaneous elevation of these biomarkers emphasizes the need for a detailed and prompt diagnostic approach, especially for infants deemed to be at higher risk. Furthermore, it highlights the essential nature of swift intervention to lessen the severity and advancement of myocarditis, ultimately enhancing clinical outcomes for these at-risk patients.

Presently, the specific processes that contribute to cardiac issues in NLE are not fully understood. The detection of anti-Ro52 antibodies is often linked with heightened sensitivity to ultraviolet (UV) light and the onset of congenital heart block (CHB) in those identified with NLE (4). These antibodies considerably enhance the likelihood of second- and third-degree atrioventricular block (4). There is a significant lack of statistical support that shows anti-La antibodies by themselves heighten the risk of CHB more than anti-Ro antibodies do (32). Nevertheless, the occurrence of CHB is significantly more frequent when both anti-Ro and anti-La antibodies are present, and this frequency further increases in instances where all three antibodies (anti-Ro52, anti-Ro60, and anti-La) are found (33, 34). Experimental studies have revealed that the death of cardiomyocytes reveals Ro and La antigens on their surfaces, which are then targeted by maternal anti-Ro and anti-La antibodies (32). This interaction leads to the creation of immune complexes that hinder apoptosis and trigger an inflammatory response. In living organisms, opsonization might transform a non-inflammatory process into an inflammatory one, as macrophages release TNFα and TGF-β, which ultimately encourage fibroblast differentiation and scar tissue formation. This sequence of events has the potential to result in myocarditis, fibrosis, and heart block, which can eventually lead to heart failure (35, 36). Maternal antibodies that penetrate the placental barrier attack fetal antigens during apoptosis, resulting in immune complex formation and an inflammatory response that negatively influences fetal cardiac tissue.

In this case analysis, the patient received a successful treatment plan that integrated intravenous immunoglobulin (IVIG) with methylprednisolone, resulting in notable improvements. IVIG has demonstrated considerable therapeutic advantages in treating pediatric systemic lupus erythematosus (SLE), significantly increasing the efficacy of SLE treatments, particularly in severe cases (37, 38). Guidelines provided by European authorities clearly support the use of IVIG for all severe lupus erythematosus situations (38). Anti-SSA and anti-SSB antibodies not only act as key pathogenic agents in neonates with NLE but are also essential for the progression of SLE. The fundamental mechanisms behind both SLE and NLE are rooted in immune cross-reactions involving antinuclear antibodies (ANA) and the body's tissues, with secondary tissue damage manifesting similarly in both disorders, indicating possible commonalities in their underlying etiologies. IVIG serves as a neutralizing antibody capable of counteracting autoantibodies (39). When there is a significant influx of exogenous IgG into the human body within a short timeframe, it alters the equilibrium of antigens and antibodies in the blood, thereby diminishing the formation of antigen-antibody immune complexes. Furthermore, individual clinical case reports have indicated that IVIG may yield specific therapeutic benefits for NLE and assist in managing NLE myocarditis, especially when used alongside pacemaker therapy (19). Consequently, further research is required to assess the efficacy of IVIG combined with methylprednisolone in treating neonatal lupus complicated by severe myocarditis and to refine the treatment strategy for this complication.

## Data Availability

The original contributions presented in the study are included in the article/Supplementary Material, further inquiries can be directed to the corresponding author.
